# *Coix* Seed Extract Attenuates Glycolipid Metabolism Disorder in Hyperlipidemia Mice Through PPAR Signaling Pathway Based on Metabolomics and Network Pharmacology

**DOI:** 10.3390/foods14050770

**Published:** 2025-02-24

**Authors:** Min Wang, Tianming Yang, Yongjing Xiang, Junxiao Pang, Yao Wang, Dali Sun

**Affiliations:** 1 The Key Laboratory of Environmental Pollution Monitoring and Disease Control, Ministry of Education, School of Public Health, Guizhou Medical University, Guiyang 561113, China; wm1999929@163.com (M.W.); tianmingyang27@163.com (T.Y.); xyj4ns@163.com (Y.X.); wygzykdx@163.com (Y.W.); 2 College of Food Science and Engineering, Guiyang University, Guiyang 550005, China; pjx111983@163.com

**Keywords:** *coix* seed extract, hyperlipidemia, metabolomics, gut microbiota, network pharmacology

## Abstract

Hyperlipidemia is characterized by a high level of blood lipid which poses a serious threat to human health. *Coix* seed is a traditional crop of medicine and food homology with a wide range of pharmacological actions. To make clear the attenuation effect of *coix* seed against hyperlipidemia, low and high doses of *coix* seed extract (CSE) were orally administered to hyperlipidemia model mice developed by high-fat diet (HFD). Our results showed that CSE notably improved liver pathological injury, and oxidative stress, and declined the levels of glucose and lipid in hyperlipidemia mice. Liver metabolomics showed that lipid-related metabolites notably decreased, and pathways of glycolipid metabolism were seriously affected by CSE intervention. Moreover, 16S rRNA sequencing revealed that CSE treatment notably increased the diversity of gut microbiota. Meanwhile, the microbiota with the function of regulating intestinal balance as well as relieving obesity and nervous diseases significantly enhanced while harmful flora notably decreased after CSE intervention. The results of network pharmacology and molecular docking indicated that the PPAR signaling pathway may be the core path of anti-hyperlipidemia for *coix* seeds. RT-qPCR further verified that the expression levels of genes from the PPAR pathway notably changed by CSE treatment with fat synthesis genes significantly decreased while lipolysis genes notably enhanced. Therefore, *coix* seed might be a potential candidate for the treatment of hyperlipidemia.

## 1. Introduction

Hyperlipidemia is marked by elevated levels of triglycerides (TGs) or cholesterol, which can be associated with various complications and may be caused by factors such as genetics, HFD, obesity, and diabetes [1]. Chronic hyperlipidemia might result in arteriosclerosis, coronary heart disease (CHD), cerebral infarction, cardiovascular infarction, visual impairment, and so on [2]. It also increases the likelihood of developing high blood pressure, pancreatitis, hepatitis, and Alzheimer’s disease (AD) [3]. Around 2008, the occurrence rate of hyperlipidemia was about 25% around the world and led to 17 million people dying each year due to hyperlipidemia and its related diseases like cerebrovascular diseases (CVDs) and atherosclerosis [4]. Currently, statins, bates, and niacin are the most effective and commonly used medications for treating hyperlipidemia, although they may have adverse effects on the liver or other organs as a result [5]. Therefore, a safe and effective method to prevent and control hyperlipidemia was urgently needed.

*Coix* seed (*Coix lacryma-jobi*) is a homologous plant of medicine which holds an important place in the cultural practices of numerous Asian communities. It serves not only as a dietary staple in traditional cuisines but also symbolizes health and prosperity. Due to its high nutritional and medicinal values, the demand for *coix* seed and its extracts increased which effectively integrated traditional wisdom with modern scientific research [6]. *Coix* seed extracts, including coixenolide, coixol, and polysaccharides, were obtained from the seeds of the *coix* plant by using water, ethanol, methanol, or other solvent extracts [7]. Modern pharmacological studies showed that *coix* seed has various medicinal properties such as anti-cancer, antioxidants, anti-obesity, anti-inflammatory, anti-hypertension, endocrine regulation, and cardiovascular protection [8]. Ethanolic or water extracts of *coix* seed attenuated hepatic steatosis and inflammation inhibited lipogenesis and alleviated nonalcoholic fatty liver disease (NAFLD), along with its associated conditions in mice on an HFD [9]. Polysaccharides from *coix* seeds have a hypoglycemic effect and can improve diabetes complications in diabetic mice [10,11]. *Coix* seed oil (CSO) mitigated apoptosis in PANC-1 pancreatic cancer cells by modulating mitochondrial activity and associated apoptotic factors that are significantly linked to the Phosphatidylinositol 3-kinase/protein kinase B (PI3K/AKT) signaling pathway [12]. The findings from the dietary study indicated that a daily intake of 60 g of dehulled adlay notably reduced fat mass levels, total cholesterol (TC), triglycerides (TGs), and the inflammatory markers Tumor Necrosis Factor-alpha (TNF-α), Interleukin 6 (IL-6), and Interleukin 10 (IL-10), among adults who are overweight or obese [13]. Polyphenol from *coix* seed significantly lowered bodyweight, liver weight, contents of TC and TGs in the liver, as well as glucose levels in serum by activating phosphorylated-AMP-activated protein kinase/phosphorylated-Acetyl-CoA carboxylase (p-AMPK/p-ACC) pathway in HFD mice [14]. These biological activities made it a valuable natural resource in traditional medicine. However, whether *coix* seed could alleviate hyperlipemia in HFD-induced mice and the mechanism of action remains unclear.

The integration of metabolomics with gut microbiota analysis has emerged as a prevalent method for studying mechanisms [15]. Metabolomics is used to identify varied metabolites and altered metabolic pathways. Analyzing the gut microbiome may aid in comprehending the shifts in the composition and abundance of gut flora, thereby uncovering the connection between microbiota and the health of the host. Metabolites of intestinal flora such as branched-chain amino acids and short-chain fatty acids (SCFAs) were considered the important mediators of intestinal flora and host health playing important role in immunomodulation, detoxification, anti-tumor, anti-hyperlipemia, etc [16]. The results of metabolomics and gut microbiota showed that selenium-enriched kiwifruit significantly increased the abundance of beneficial bacteria in the colon and improved hyperlipidemia by regulating lipid-related metabolic pathways in HFD mice [17]. Flavonoids and dietary fiber from Shatianyu (*Citrus grandis* L. *Osbeck*) have also been indicated to help reduce hyperlipidemia through the modulation of gut microbiota and the production of butyrate, which in turn affects lipid metabolism in mice fed a high-fat diet [18].

Network pharmacology is primarily utilized to examine the networks of biological systems in order to select specific signaling nodes for multi-target molecules. This approach has been effectively employed to discover potential active compounds in traditional Chinese medicine [19]. Molecular docking serves as an important tool for analysis of the interaction between the proposed functional component and primary target which could be applied to verify results obtained from network pharmacological analysis. Based on these methods, 42 potential targets for oleanolic acid (OA) and obesity were selected. PPARγ and PPAR signaling pathways were the main target and pathway, respectively [20]. A total of 55 interactions were identified between hyperlipidemia and Yinchen Wuling powder using network pharmacology and molecular docking analysis. The key binding targets were PTGS2 protein with quercetin, isorhamnetin, and taxifolin [21]. Qinlian Hongqu decoction (QLHQD) notably reduced hyperlipidemia in rats on an HFD via the IRE1-α/IKKB-β/NF-κB signaling pathway, as demonstrated by both network pharmacology analysis and experimental validation [22]. Hence, the potential molecular mechanisms associated with the treatment of different diseases through phytochemicals may be uncovered by utilizing network pharmacology and molecular docking techniques.

The combination of network pharmacology with metabolomics and gut microbiota could promote the understanding of the underlying mechanism of disease development, providing powerful tools and methods for the development of new medicine. A network pharmacology study showed that arecoline and G-protein-coupled receptor signaling pathways might be the key targets for areca nut addition. Metabolome analysis additionally revealed that arecoline and bile acids may serve as potential targets in the development of addictive behavior associated with areca nut chewing [23]. The combined analysis indicated that unprocessed rhubarb reduced the area of cerebral infarction and the inflammatory response, enhanced intestinal barrier function, and raised the level of Firmicutes. This study investigated the mechanism by which unprocessed rhubarb acts against ischemic stroke through the microbiota-gut–brain axis [24]. However, the mechanism of action for the preventive effect of CSE on hyperlipidemia was still unclear which could be declared by the mentioned technologies.

Consequently, the objective of our research was to uncover the possible therapeutic benefits of CSE in mice experiencing hyperlipidemia induced by a high-fat diet. Non-targeted metabolomics combined with 16S rRNA sequencing analyses were introduced to identify the differential metabolites, pathways, and flora. Additionally, molecular docking and network pharmacology were utilized to identify the active sites linking the ingredients and their respective targets. The results of this study could systematically clarify the underlying mechanism of CSE against hyperlipidemia.

## 2. Materials and Methods

### 2.1. Coix Seed Extraction

According to the previous study [25], CSE was prepared by the water extraction method. The dried and matured *coix* seed was selected and fully crushed. After that, a reflux extraction was conducted with water and for 3 recyclings. The extracts were subsequently concentrated using a rotary evaporator for a duration of 2 h and then dried in a vacuum drying furnace at a temperature of 80 °C to yield the solid CSE, which was stored at 4 °C for use in experiments. The detailed component analysis for CSE was shown in Supplementary Materials.

### 2.2. Animal Experiment

The animal experiment was conducted in strict accordance with the ethical requirements authorized by the Animal Ethics Committee of Guizhou Medical University (SCXK20230002). SPF-grade male C57BL/6J mice (4 weeks old) were purchased from Changsha Tianqin Biotechnology Co., Ltd. (Changsha, China). Mice were maintained in an animal facility at a temperature of 25 ± 2 °C, with a humidity range of 50–60%, and subjected to 12 h cycles of natural light and darkness. After 1-week adaptation, mice were randomly divided into a control group (n = 10) and a hyperlipidemia group (HFD, n = 30).

During the experiment, mice in the control group were fed with basal diet consisting of corn, soybean meal, wheat submeal, fish meal, soybean oil, stone powder, calcium bicarbonate, vitamins, and mineral elements while mice in the hyperlipidemia group were fed with HFD consisted of basal diet, fructose, casein, lard, osesame oil, and cholesterol. The detailed diet composition percentages for the control and HFD groups are shown in Table S1. After a period of 8 weeks, blood samples were taken from the tail tips of the mice, and measurements of lipid indices were conducted. Serum was collected by taking blood from the tip of the mouse’s tail. The levels of TC, TGs, LDL-C, and HDL-C in the serum were detected using the corresponding kits (Nanjing Jiancheng Bioengineering Institute, Nanjing, China) and a microplate reader (Thermo Fisher Scientific Inc., Waltham, MA, USA). The notable increase in TC, TGs, and LDL-C contents and decrease in HDL-C level in the HFD group compared to the control group indicated that the hyperlipidemia mouse model was successfully constructed. After successful modeling, 30 hyperlipidemia mice were randomly divided into the model group, low-dose group, and high-dose group (n = 10) which were orally exposed to CSE (dissolved and diluted by pure water) with the dosage of 0, 1.3, and 5.2 g/kg⋅*bw*, respectively. The given dose of CSE was calculated by the body surface area conversion algorithm according to the general human consumption dose originally published in the 2020 edition of Chinese Pharmacopeia. For 8 weeks, both the control and model groups of mice received an identical daily volume of normal saline. At the end of the intervention, mice were killed by cervical dislocation. Samples of blood were collected from the eyeballs as well as from liver tissues, and the contents of the cecum were gathered and weighed. These samples were then rapidly frozen in liquid nitrogen and subsequently stored at −80 °C for further examination.

### 2.3. Growth Indexes and Pathological Analysis

Each week throughout the intervention, the weight of the mice and their food consumption were recorded. In drug toxicity studies or chemical substance exposure experiments, the ratio of liver weight to bodyweight (liver-to-bodyweight ratio) is one of the sensitive indicators for evaluating whether the liver has been damaged. The ratio of liver weight to bodyweight was measured on the last day of the experiment. For the analysis of pathology, liver tissue samples were preserved in 4% paraformaldehyde for a duration of 24 h, subsequently embedded in paraffin, and sliced into sections of 5 μm thickness. These sections were then treated with a 10% (*v*/*v*) formaldehyde solution, stained using H&E, and examined under an Olympus microscope. Concurrently, the liver sections, each 10 μm thick, were stained using a modified oil red O solution and subsequently re-stained with hematoxylin. The droplets of lipids were observed under a microscope (Nikon Corporation, Tokyo, Japan). The remaining liver samples were applied for antioxidant enzyme activity and biochemical indexes analysis.

### 2.4. Determination of Blood Glucose Level

The fasting blood glucose (FBG) test was determined once a week during the 8 weeks of CSE treatment. After a 12 h overnight fast, blood was drawn from the mice tails and glucose levels were assessed using a Sannuo GA-3 glucose meter (Nuo Biosensor Co., Ltd., Changsha, China). For OGTT, mice were fasted overnight for 12 h the day before the end of the experiment. After that, glucose solution (2.0 g/kg·bw) was given by an oral administration. Blood samples were collected at 0, 30, 60, 90, and 120 min after glucose load, and levels of glucose were measured. The glucose area under the curve (AUC) was determined using the trapezoid rule.

### 2.5. Determination of Antioxidant Enzyme Activity and Biochemical Index

The activities of antioxidant enzymes SOD, CAT, and MDA were determined to evaluate the effects of CSE on the liver. Liver tissues were homogenized in liquid nitrogen and diluted with normal saline (1:9 *w*/*v*). After centrifugation at 2500 rpm for 10 min, the supernatant was collected for further analysis. In addition, AST and ALT related to liver function were also detected according to the instructions of the corresponding kit (Nanjing Jiancheng Bioengineering Institute, Nanjing, China). Serum samples were obtained from blood samples which were placed overnight at 4 °C first, centrifuged at 3000 rpm/min for 10 min, and then the supernatant was collected. Levels of TC, TGs, LDL-C, and HDL-C in serum and liver were detected to assess the alleviation effect of CSE on lipid-related indicators, which were also measured by the corresponding kit (Nanjing Institute of Jiancheng Bioengineering, Nanjing, China) and determined by a microplate reader (Thermo Fisher Scientific, USA).

### 2.6. Metabolomics Analysis

The previous study method [26] was used for metabolomics analysis, 20 mg liver samples were weighed, and extracted with 400 μL 70% methanol/water solution with internal standard (dichlorophenylalanine) added first. Samples were agitated at 1500 r/min for 5 min, chilled on ice for 15 min, and centrifuged at 4 °C and 12,000 r/min for 10 min. The supernatant was then transferred to a new tube and stored at −20 °C for 30 min. After a final centrifugation at 4 °C and 12,000 r/min for 3 min, the supernatant was collected for analysis. Six quality control (QC) samples of equal volume were prepared by pooling supernatants from all samples to assess data stability, accuracy, and reliability. All samples were detected on an LC-30A ultra-high performance liquid chromatography (Shimadzu Corporation, Kyoto, Japan) equipped with a triple TOF 6600+ mass spectrometer (AB Sciex, Framingham, MA, USA). Samples were analyzed using a Waters ACQUITY Premier HSS T3 column (Waters Corporation, Milford, MA, USA) (1.8 µm, 2.1 mm × 100 mm) with a mobile phase consisting of 0.1% formic acid in water (A) and 0.1% formic acid in acetonitrile (B). The column was maintained at 40 °C, with a flow rate of 0.4 mL/min and an injection volume of 4 µL. The initial gradient elution was 95% phase A and 5% phase B, changed to 80~40% phase A and 20~60% phase B at 2–5 min, and then 1% phase A and 99% phase B at 6.0–7.5 min, finally 95% phase A and 5% phase B at 7.6–10 min. The ionization voltage and ion source temperature were set at 5 kV and 550 °C for positive mode, and −4 kV and 450 °C for negative mode. Mass ranges for MS1 and MS2 were set from 25 to 1000 *m*/*z* and from 50 to 1000 *m*/*z*, respectively. ProteoWizard converted the raw mass spectrometry data into mzXML format, and XCMS was applied for peak extraction, alignment, and retention time correction.

### 2.7. Gut Microbiota Analysis

The composition and abundance changes in gut microbiota by CSE intervention were determined by 16S rRNA sequencing. Cecal contents (500 mg) were collected and quickly frozen in liquid nitrogen. Genomic DNA was isolated using the Omega Bio-tek Stool DNA Kit (Omega Bio-Tek, Norcross, GA, USA) according to the manufacturer’s protocol. The bacterial 16S rRNA gene’s V3-V4 region was amplified via PCR with primers 338F (5′-ACTCCTACGGGGGGCAGCA-3′) and 806R (5′-GGACTACHVGGGTWTCTAAT-3′). Equal amounts of PCR products, based on concentration, were pooled and purified using 2% agarose gel electrophoresis (Thermo Fisher Scientific, Waltham, MA, USA). After that, products were recovered by gel recovery kit (Qiagen, Hilden, Germany) for the target bands. Sequence execution was conducted using the Illumina Novaseq6000 platform (Illumina, San Diego, CA, USA).

### 2.8. Network Pharmacology

The platforms and databases system of TCMSP (https://old.tcmsp-e.com/tcmsp.php, accessed on 1 February 2025), PubChem (https://pubchem.ncbi.nlm.nih.gov/, accessed on 1 February 2025), and Swiss Target Prediction (http://swisstargetprediction.ch/, accessed on 1 February 2025) were applied to extract the active ingredients and their gene names of *coix* seed. By searching the keywords of “hyperlipidemia” and “Homo sapiens”, hyperlipidemia related targets were obtained based on the databases of OMIM (https://omim.org/, accessed on 1 February 2025), TTD (https://db.idrblab.net/ttd/, accessed on 1 February 2025), and GeneCards (http://www.genecards.org/, accessed on 1 February 2025). The target intersection was conducted by using Bioinformatics (https://www.bioinformatics.com.cn/, accessed on 1 February 2025) to search for the overlapping genes related to hyperlipidemia and components of *coix* seed. Moreover, the Venn diagrams were obtained for the visual representation of results, and the network diagram of “component-target-potential disease” was constructed using Cytoscape 3.7.1 software. The PPI network was constructed and visualized using the String Protein Interaction Network database (https://cn.string-db.org/, accessed on 1 February 2025) and the obtained data were applied for PPI network topology analysis based on Cytoscape 3.7.1 software. GO functional enrichment and KEGG pathway analyses were performed using the DAVID database (https://david.ncifcrf.gov/, accessed on 1 February 2025) to identify key targets related to *coix* seed and hyperlipidemia.

### 2.9. Molecular Docking

The target protein structures were obtained from the PDB database (https://www.rcsb.org/, accessed on 1 February 2025), and the 3D structures of the targeted components of the *coix* seed were retrieved from the TCMSP platform (https://old.tcmsp-e.com/tcmsp.php, accessed on 1 February 2025). Small molecules and water were removed from the target protein using Pymol 3.1.3, and its charge was calculated with AutoDock 4. The ligand’s rotation bond and docking box were defined, followed by rigid docking. Results were visualized and analyzed in Pymol.

### 2.10. RT-qPCR Analysis

Based on network pharmacology and molecular docking results, 7 PPAR signaling pathway genes peroxisome proliferator-activated receptor alpha (*Pparα)*, peroxisome proliferator-activated receptor gama (*Pparγ)*, recombinant liver X receptor alpha (*Lxrα)*, glucose transporter 4 (*Glut4)*, stearoyl-CoA desaturase 1 (*Scd1)*, *Hmgcr*, and cholesterol 7α-hydroxylase (*Cyp7a1)* that related to glucose and lipid metabolism were detected to declare the mechanism of CSE on hyperlipidemia mice induced by HFD. Total RNA from liver samples was isolated using TRIzol reagent (TaRaKa, Tokyo, Japan), with concentration and purity measured by a NanoDrop spectrophotometer (Thermo Fisher Scientific, USA). Subsequently, 1.0 μg of RNA was reverse-transcribed into cDNA using a kit (Takara, Toky, Japan). Real-time PCR was performed with a qPCR SYBR Green Master Mix Kit (Takara, Japan), using β-actin as the internal reference. All gene primers were synthesized by Shenggong Bioengineering Co., Ltd. (Shanghai, China). The primer sequences are listed in Table S2. Gene expression levels were quantified using the 2^−ΔΔCt^ method with the comparative CT approach. Each sample was analyzed in triplicate.

### 2.11. Statistical Analysis

Data were analyzed using one-way ANOVA and Tukey’s test, expressed as mean ± SD (X ± SD). Statistical analyses were conducted using GraphPad Prism 8 (La Jolla, CA, USA). Significant differences were labeled as *p* < 0.05 (* or ^#^), *p* < 0.01 (** or ^##^), *p* < 0.001 (*** or ^###^), and *p* < 0.0001 (**** or ^####^). For metabolomics, *p*-values were determined using a Student’s *t*-test, with VIP > 1 and *p* < 0.05 identifying differential metabolites. For gut microbiota, The Kruskal–Wallis test was used to evaluate the α diversity (Shannon and Chao1 indices) and inter-group differences in intestinal flora in the R software version v4.2.0. *p* value was calculated using a Student’s *t*-test.

## 3. Results

### 3.1. Chemical Composition of CSE

Totally 195 chemical components from CSE were identified including 59 flavonoids, 40 alkaloids, 32 phenols, 17 fatty acids and their esters, 12 coumarins, 11 amino acids and their derivatives, 9 triterpenes, 8 sterols, 7 nucleotides and their derivatives. The top 10 components were oleic acid, L-phenylalanine, L-pipecolic acid, pimelic acid, 5-oxoproline, palmitic acid, 6-aminocaproic acid, L-valine, beta-sitosterol, stigmasterol which mainly belonged to the classes of fatty acyls, amino acid and derivatives, steroids and steroid derivatives (Table S3).

### 3.2. Effects of CSE on Bodyweight in Hyperlipidemia Mice

**Figure 1:**
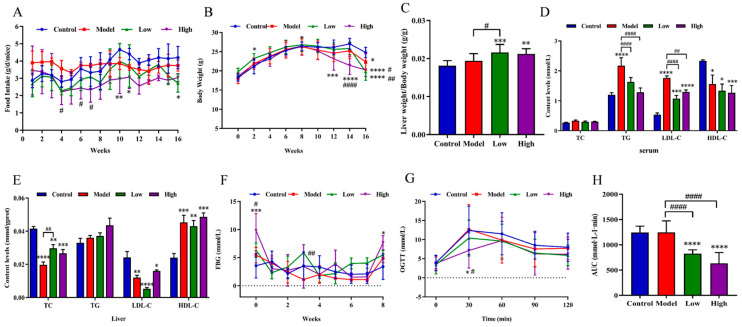
Effects of CSE intervention on body indexes as well as lipid and glucose levels in hyperlipidemia mice. Food intake (**A**), bodyweight (**B**), liver weight/bodyweight ratio (**C**), lipids in serum (**D**) and liver (**E**), FBG (**F**), OGTT (**G**), and the area under the curve of glucose (**H**). Data were shown as means ± RSD. * indicates the significance between the CSE-treated group and the control group. # indicate the significance between the CSE-treated group and model group (*^,#^ *p* < 0.05, **^,##^ *p* < 0.01, *** *p* < 0.001, ****^,####^ *p* < 0.0001).

During the experiment, the food intake and bodyweight of the mice were monitored. As shown in Figure 1A, the food intake at weeks 4, 6, and 7 notably decreased in the high-dose group compared to the model group (*p* < 0.05), and food intake at weeks 10, 11, and 16 also significantly declined at CSE treatment groups compared to the control group (*p* < 0.01, *p* < 0.05, *p* < 0.05). Overall, the food intake of CSE-treated groups was relatively lower than control and model groups, especially in the high-dose group. This might be because of that CSE intake provided energy for mice and then reduced food intake. The bodyweight of mice generally increased before week 8 while notably decreased by CSE intervention. A significant decrease in bodyweight was observed in the high-dose group compared to the control group in the last 3 weeks (*p* < 0.0001). Meanwhile, a remarkable decline in bodyweight was also detected in the high-dose group for weeks 14 and 16 (*p* < 0.0001, *p* < 0.01) and in the low-dose group for week 16 (*p* < 0.05) compared to the model group (Figure 1B). The change tendency of bodyweight was basically consistent with the amount of food intake. The ratio of liver weight to bodyweight (liver coefficient) notably increased after CSE intervention compared to control and model groups probably due to the decrease in bodyweight of mice in CSE-treated groups (Figure 1C). The detailed values of food intake, bodyweight, FBG, and OGTT were present in Tables S4–S7. These results suggested that CSE intervention reduced bodyweight of hyperlipidemia mice, thus preventing obesity.

### 3.3. Effects of CSE on Lipids and Glucose Levels in Hyperlipidemia Mice

Levels of lipids were notably disturbed by CSE intervention in hyperlipidemia mice. HFD notably increased TG levels in serum (*p* < 0.0001) while CSE intervention remarkably decreased TG content at low and high-dose groups (*p* < 0.0001). Levels of LDL-C notably rose in the model group compared to the control group while significantly decreased after CSE treatment (*p* < 0.0001). However, the contents of HDL-C in the model, low-, and high-treated groups notably decreased compared to the control group (*p* < 0.05, *p* < 0.05, *p* < 0.001) (Figure 1D). In the liver, contents of TC notably decreased in the model, low-, and high-dose groups (*p* < 0.0001, *p* < 0.01, *p* < 0.001) compared to the control group while low doses of CSE treatment notably increased the TC levels compared to the model group. Similarly, levels of LDL-C significantly declined in the model, low-, and high-dose groups (*p* < 0.01, *p* < 0.0001, *p* < 0.05) compared to the control group. HDL-C contents in the model and two CES groups significantly raised compared to the control group (*p* < 0.001, *p* < 0.01, *p* < 0.001) (Figure 1E). The detected contents of TC, TGs, LDL-C, and HDL-C in serum were relatively higher than those in the liver. This may be because that serum is the place where lipids are used, stored, and transported. Our results suggested that high levels of lipids were detected in blood-forming hyperlipidemia in mice while CSE intervention remarkably alleviated lipid levels.

A notable increase in FBG level was observed in the high-dose group compared to the control group at weeks 0 and 8 (*p* < 0.001, *p* < 0.05). Compared to the model group, significant differences were observed at week 0 in the high-dose group and week 3 in the low-dose group (*p* < 0.05, *p* < 0.01) (Figure 1F). For the OGTT experiment, levels of blood glucose significantly increased and reached their peak value within 30 min in the control, model, and low-dose groups while peaked at 1 h in the high-dose group after oral administration of glucose which indicated that CSE intervention notably increased the glucose tolerance in mice and remarkably decreased glucose levels in the high-dose group compared to control and mode groups (*p* < 0.05) (Figure 1G). The glucose area under the curve (AUC) was significantly lower in CSE-treated groups compared to control and model groups (*p* < 0.0001), respectively, also indicating that CSE significantly improved glucose intolerance in hyperlipidemia mice (Figure 1H).

### 3.4. Effects of CSE on Liver Function in Hyperlipidemia Mice

**Figure 2:**
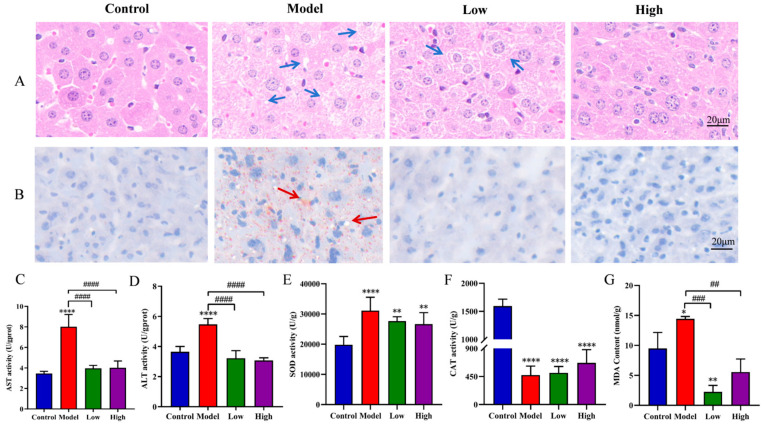
Effects of CSE on liver function in hyperlipidemia mice. The pathological changes in the liver by CSE intervention (**A**), oil red O staining (×20) (**B**) (Note: blue and red arrows indicate vacuole and inflammation, and lipid droplets), AST content (**C**), ALT content (**D**), activities changes in SOD (**E**), CAT (**F**) and MDA content (**G**). Data were shown as the means ± RSD (n = 5). * indicates the significance between the CSE-treated group and the control group. # indicates the significance between CSE-treated group with model group (* *p* < 0.05, **^,##^ *p* < 0.01, ^###^ *p* < 0.001, ****^,####^ *p* < 0.0001).

The results of histopathological examination for liver tissues showed that severe hepatocyte swelling and vacuole were observed in the model group while this phenomenon was alleviated after a low dose of CES treatment and completely disappeared in the high-dose group, showing its protective effect on hyperlipidemia mice liver (Figure 2A). Oil red O was used to evaluate fat droplets that accumulated in the liver. Obvious elliptical fat vacuoles and fat droplets in the model group were observed compared to the control group while the number and percentage of fat vacuoles and fat droplets notably reduced after CSE intervention, especially in the high-dose group (Figure 2B). The activities of liver function indexes ALT and AST significantly increased in the model group compared to the control group (*p* < 0.0001) while both reduced to normal levels after CSE treatment. These results indicated that CSE intervention could reduce ALT and AST contents in hyperlipidemia mice and protect liver function (Figure 2C,D). SOD activity significantly increased in the model, low-dose, and high-dose groups compared to the control group (*p* < 0.0001, *p* < 0.01, *p* < 0.01) while no difference was observed among the model group and CES-treated groups (Figure 2E). A notable decrease in CAT activity was determined in model, low-, and high-dose groups compared to the control group (*p* < 0.0001, *p* < 0.0001, *p* < 0.0001) with no obvious difference among model group and CES-treated groups (Figure 2F). MDA is an important index that reflects the degree of lipid peroxidation in cell membranes. Our results showed that the content of MDA remarkably decreased in low and high doses of CSE treatment compared to the model group (*p* < 0.001, *p* < 0.01) and even lower than the control group at the low-dose group (*p* < 0.01) (Figure 2G). The results suggested that HFD could impair liver function in hyperlipidemia mice which could be relieved by CSE intervention.

### 3.5. Effects of CSE on Metabolic Profiles in Hyperlipidemia Mice

Liver metabolomics was conducted to elucidate the alleviation of CSE in hyperlipidemia mice. Orthogonal partial least squares discriminant analysis (OPLS-DA) score plots revealed clear separation among the control, model, low-dose, and high-dose groups (Figure S1A) and good predictive ability was observed with R^2^Y(perm) = 0.933, Q^2^(perm) = 0.438 for model vs. low group, and R^2^Y(perm) = 0.986, Q^2^(perm) = 0.467 for model vs. high group (Figure S1B,C). The R^2^Y value of the original model approached 1, suggesting the model effectively explained the data. Q^2^ values suggested that CSE-treated groups obviously separated from the model group. These results declared that CSE exposure significantly changed the metabolic profiles in the liver of hyperlipidemia mice.

**Figure 3:**
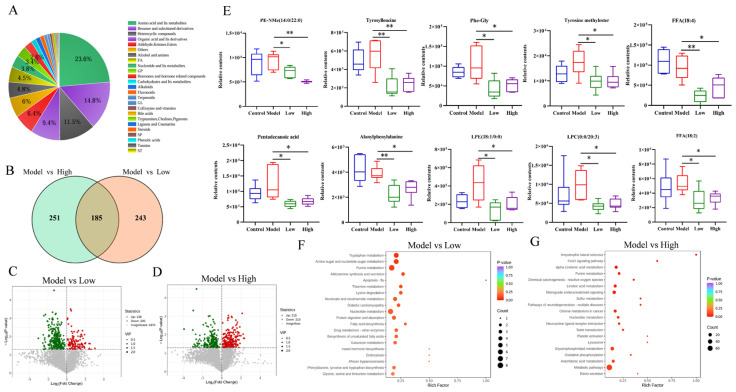
Effects of CSE intervention on metabolic profiles of the liver in hyperlipidemia mice. Species ratio of changed metabolites (**A**), Venn analysis of differential metabolites (**B**), volcano plots for metabolites under the conditions of VIP > 1 and *p* < 0.05 for model vs. low-dose group (**C**), and model vs. high-dose group (**D**) (Note: green color indicates significantly down-regulated metabolites, red color indicates significantly up-regulated metabolites), the contents of shared metabolites by low and high doses of CSE intervention (**E**), KEGG pathway enrichment of the changed metabolites for model vs. low-dose group (**F**) and model vs. high-dose group (**G**). Data were shown as the means ± RSD (n = 6). * indicates the significance between CSE-treated group with model group (* *p* < 0.05, ** *p* < 0.01).

Totally 5785 metabolites were detected by UPLC-MS/MS which could be classified into 24 subgroups. The highest contents of metabolites belonged to amino acid and its metabolites (23.6%), followed by benzene and substituted derivatives (14.8%), heterocyclic compounds (11.5%), organic acid and its derivatives (9.4%), and lipid-like molecules (8.2%) (Figure 3A). Potential biomarkers were identified using criteria of VIP > 1 and *p* < 0.05. The volcano map represented that 428 metabolites (138 up-regulated and 290 down-regulated) were significantly disturbed in the low-dose group and 436 metabolites (218 up-regulated and 218 down-regulated) were notably changed in the high-dose group compared to the model group (Figure 3B–D). Among these metabolites, 185 were shared by low- and high-dose exposure. The top 10 differential metabolites contained 6 lipid PE-NMe (14:0/22:0), LPE(18:1/0:0), LPC(0:0/20:3), FFA(18:4), FFA(18:2), and pentadecanoic acid as well as four amino acids and its metabolites Phe-Gly, tyrosine methylester, alanylphenylalanine, and tyrosylleucine. Compared to the model group, levels of these 10 differential metabolites were notably reduced after low and high doses of CSE intervention (*p* < 0.05, *p* < 0.01) (Figure 3E). Pathway analysis was performed with KEGG enrichment analysis to gain insight into potential biological pathways associated with the identified biomarkers. Four dominant metabolic pathways were disturbed in the low-dose group including purine metabolism, amino sugar, and nucleotide sugar metabolism, tryptophan metabolism, and nucleotide metabolism (Figure 3F). In addition, metabolic pathways of glycerophospholipid metabolism, retrograde endocannabinoid signaling, and linolenic acid metabolism were also seriously affected in the high-dose group compared to the control group which were mainly related to lipid metabolism and nervous system (Figure 3G).

### 3.6. Effects of CSE on Gut Microbiota in Hyperlipidemia Mice

**Figure 4:**
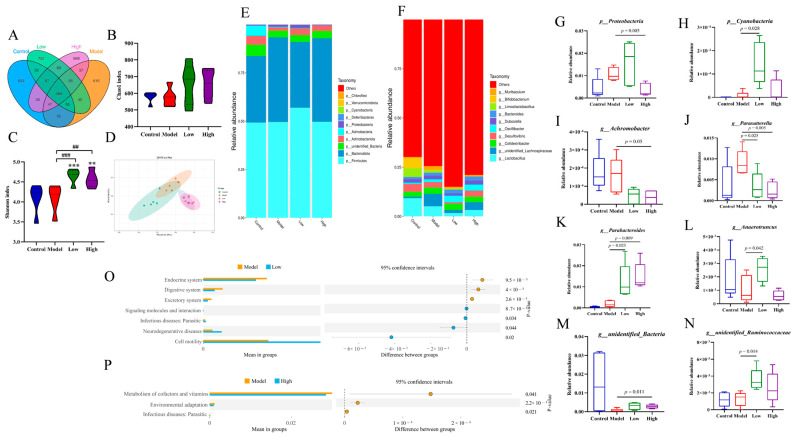
Effects of CSE intervention on intestinal flora in cecum contents. Venn diagram of detected ASVs (**A**), the indexes of Chao 1 (**B**) and Shannon (**C**), PCoA plot (**D**), flora composition at phylum level (**E**) and genus level (**F**), differential bacteria at phylum and genus levels (**G**–**N**), function prediction analysis based on *t*-test for model vs. low-dose group (**O**) and model vs. high-dose group (**P**). Data were shown as the means ± RSD. * indicates the significance between the CSE-treated group and the control group. # indicates the significance between CSE-treated group with model group (**^,##^ *p* < 0.01, ***^,###^ *p* < 0.001).

The effect of CSE on gut microbiota in hyperlipidemia mice was investigated by 16S rRNA sequencing analysis. The Venn diagram showed that low vs. model and high vs. model groups shared 549 amplicon sequence variants (ASVs) and 653, 616, 732, and 669 ASVs were unique for control, model, low-, and high-dose groups, respectively, indicating that CSE interfered with the similarity of ASV composition (Figure 4A). No obvious difference was detected in the Chao1 index among different groups, suggesting that CSE had no obvious influence on the abundance of intestinal flora (Figure 4B). However, a remarkable increase in the Shannon index was observed after low and high doses of CSE intervention compared to control and model groups, respectively (*p* < 0.001, *p* < 0.01), indicating that CSE significantly enhanced the diversity of intestinal flora in hyperlipidemia mice (Figure 4C). The PCoA plot showed that gut microbiota in control and model groups were completely separated from CSE-treated groups (Figure 4D). These findings indicate that CSE treatment significantly altered the gut microbiota composition in hyperlipidemic mice.

To further investigate changes in microbial communities, the taxonomic composition and relative abundance of each taxon were analyzed. At the phylum level, the predominant bacteria in all groups were *p_Firmicutes*, *p_Bacteroidota*, and *p_undefined Bacteria*. The highest abundance of *p_Firmicutes* in control, model, low, and high groups was 49.25%, 49.57%, 56.95%, and 49.67%, respectively (Figure 4E). Levels of *p_Proteobacteria* significantly decreased in the high-dose group while *p_Cyanobacteria* notably increased after CSE treatment compared to the model group (*p* = 0.005, *p* = 0.028) Figure 4G,H). At the gene level, *g_Lactobacillus*, *g_unidentified Lachnospiraceae*, and *g_Colidextribacter* were the dominant bacteria in four groups. The highest abundance of *g_Lactobacillus* in control, model, low, and high groups was 9.19%, 5.11%, 1.67%, and 3.21%, respectively (Figure 4F). The contents of *g_Achromobacter* at the high-dose group (*p* = 0.05) and *g_Parasutterella* at the low and high-dose groups notably declined compared to the model group (*p* = 0.025, *p* = 0.005) (Figure 4I,J). However, levels of *g_Parabacteroides* at low and high-dose groups (*p* = 0.025, *p* = 0.009), *g_Anaerotruncus* at low-dose group (*p* = 0.042), *g_unidentified_Bacteria* at high-dose group (*p* = 0.011), and *g_unidentified Ruminococcaceae* at low-dose group (*p* = 0.14) remarkably raised compared to model group (Figure 4K–N).

The heatmaps showed that gut microbiota notably changed after CSE treatment at phylum and genus levels compared to the control group (Figure S2A,B). According to LEfSe analysis and LDA score (≥4), a relatively high abundance of 10 taxa was detected in the low-dose group including *c_Campylobacterota*, *f_Atopobiaceae*, *s_TM7_phylum_sp_oral_clone_CW040*, *g_Parabacteroides*, *f_Prevotellaceae*, *s_Lachnospiraceae_bacterium_28-4*, *g_Helicobacter*, *s_Lactobacillus_murimus*, *c_Clostridia*, *o_unidentified_Clostridia* and high abundance of *g_unidentified_Lachnospiraceae* and *s_Lactobacillus_johnsonii* were observed in model group (Figure S2C,D). The pathways of cell motility, neurodegenerative diseases, and endocrine systems were significantly disturbed in low-dose and model groups (Figure 4O). In the model vs. high-dose group, high levels of *s_TM7_phylum_sp_oral_clone_CW040* and *g_Parabacteroides* were detected in high-dose group while high contents of *s_Lactobacillus_reuteri*, *g_Limosilactobacillus*, and *g_Parasutterella* in model group notably increased (Figure S2E,F) and the pathway of metabolism of cofactors and vitamins notably affected (Figure 4P). These results suggested that CSE intervention changed the intestinal flora composition in hyperlipidemia mice.

### 3.7. Network Pharmacological Analysis

**Figure 5:**
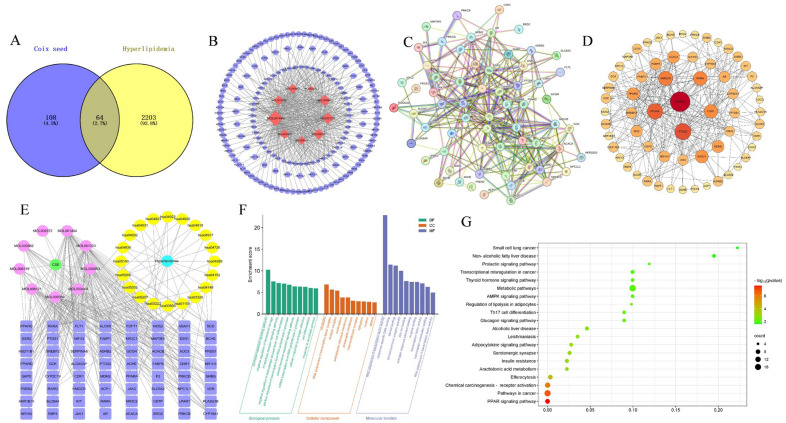
Network pharmacological analysis. Venn diagram of *coix* seed and hyperlipidemia intersection target (**A**), active ingredient-target network diagram (**B**) (Note: Red color represents the active ingredients of *coix* seed, and blue color represents targets related to hyperlipidemia), protein-protein network interaction diagram (**C**,**D**) (Note: the larger the node, the darker the color), active ingredient-disease-target-pathway network construction (**E**) (Note: purple circles are the *coix* seed active ingredients and orange circles are the pathways associated with hyperlipidemia). GO functional enrichment analysis (**F**), KEGG pathway enrichment analysis (**G**).

To investigate potential molecular action targets between *coix* seed and hyperlipidemia, network pharmacological analysis was performed. Initially, under the conditions of oral bioavailability (OB) ≥ 30% and drug similarity (DL) ≥ 0.18, 9 active ingredients were retrieved from *coix* seed based on the TCMSP database including sitosterol α1, mandenol, (6Z,10E,14E,18E)-2,6,10,15,19,23-hexamethyltetracosa-2,6,10,14,18,22-hexaene, [(2R)-2,3-dihydroxypropyl] (Z)-octadec-9-enoate, sitosterol, stigmasterol, coixenolide, 2-monoolein, and CLR. The information on active ingredients is listed in Table S8. As shown in Figure 5A,B, 108, and 2203 were screened out as the specific target genes of coix seed and hyperlipidemia, respectively, with 64 common target genes. To visually assess the effect of *coix* seed on hyperlipidemia, a PPI network consisting of 64 nodes and 291 edges was constructed using identified crossover genes, and the top six targets PPARγ, PTGS2, PPARα, HMGCR, RXRα, ESR1 were selected for analysis (Figure 5C,D). Active ingredients and cross genes were then imported into Cytoscape 3.7.1 software to construct the “active ingredient-target-disease” network. The results showed that there were 91 nodes and 258 edges in the network, and close correlations among active components, targets, and hyperlipidemia were observed (Figure 5E).

To explore the mechanism of *coix* seed in treating hyperlipidemia, GO enrichment analysis including biological process (BP), cellular component (CC), and molecular function (MF) was conducted using the DAVID database to identify therapeutic targets. Figure 5F illustrates the ten enrichment terms for BP, CC, and MF that exhibited the highest number of gene counts. BP for anti-hyperlipidemia is mainly involved in hormone-mediated signaling pathways, negative regulation of cholesterol storage, and intracellular receptor signaling pathways. CC enrichment analysis showed that chromatin, RNA polymerase II transcription factor complex, and receptor complex were the dominant action components. MF primarily included RNA polymerase II transcription factor activity, sequence-specific DNA binding, and steroid binding. KEGG enrichment analysis showed that a total of 20 related pathways were enriched which were mainly lipid metabolism-related and the most enriched pathway was the PPAR signaling pathway (Figure 5G).

### 3.8. Molecular Docking

Molecular docking was performed to predict the binding affinity between active ingredients and their binding sites. Ranking by degree from largest to smallest in the PPAR pathway and active ingredient-disease-target-pathway network, four core components sitosterol α1, CLR, sitosterol, stigmasterol, and 8 key proteins RXRα, AR, NR1H3, CYP19A1, PPARδ, PPARα, SCD, RXRγ were selected for molecular docking. As shown in Table S9, the binding energy of sitosterol α1 with PPARδ, PPARα, RXRα, AR, NR1H3, and SCD were all less than −5.0 Kcal·mol^−1^ indicated that sitosterol α1 had strong binding ability with these 6 proteins. Therefore, their molecular docking diagram was used for visual display. Sitosterol α1 bound with proteins of RXRα, AR, NR1H3, PPARδ, PPARα, and SCD through bonds of van der Waals, Alkyl, conventional hydrogen bond, and Pi-Alkyl. These proteins had strong binding ability with ASN-267, GLY-724, LEU-69, ILE-102, LYS-204, and PRO-94 through conventional hydrogen bonds, respectively (Figure S3).

### 3.9. Effects of CSE on Expression of Genes Related to Glycolipid Metabolism

**Figure 6:**
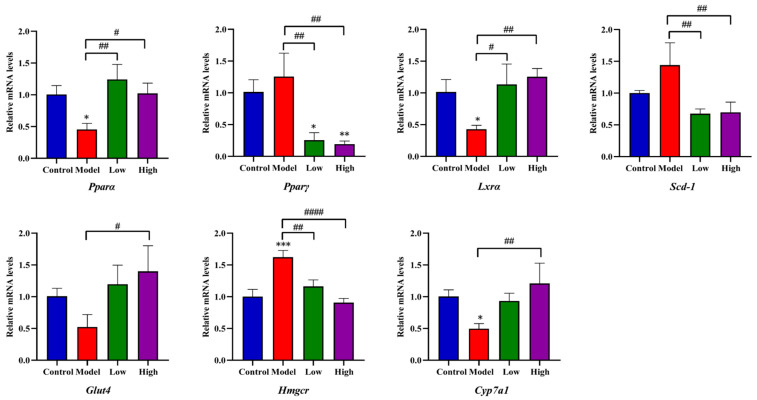
The relative mRNA expression levels of genes associated with the PPAR signaling pathway. Data were shown as the means ± RSD (n = 3). * indicates the significance between the CSE-treated group and the control group. # indicate the significance between the CSE-treated group and the model group (*^,#^ *p* < 0.05, **^,##^ *p* < 0.01, *** *p* < 0.001, ^####^ *p* < 0.0001).

The mRNA expression of genes related to glucose and lipid metabolism in the PPAR signaling pathway was analyzed. Compared to the model group, fatty acid oxidation regulation gene *Pparα* and the key gene for adipocyte differentiation *Lxrα* notably decreased after HFD exposure compared to the control group (*p* < 0.05) while significantly increasing after low and high doses of CSE treatment compared to model group (*p* < 0.01, *p* < 0.05). On the contrary, another PPAR family member *Pparγ* remarkably declined after a low and high dose of CSE exposure compared to control (*p* < 0.05, *p* < 0.01) and model group (*p* < 0.01). The key regulatory gene of monounsaturated fatty acid synthesis Scd-1 and cholesterol synthesis inhibition gene *Hmgcr* remarkably deceased after CSE treatment compared to the model group (*p* < 0.05, *p* < 0.05). Glucose transporter *Glut4* and bile acid synthesis regulation gene *Cyp7a1* significantly enhanced in high doses of CSE intervention compared to the model group (*p* < 0.05, *p* < 0.01). These results suggested that CSE improved the dysregulation of glycolipid metabolism through the PPAR signaling pathway and maintained the balance of glycolipid homeostasis in mice liver (Figure 6).

## 4. Discussion

Hyperlipidemia is a complex metabolic disease that is accompanied by atherosclerosis, myocardial infarction, and stroke, and can seriously affect organs such as the kidney, heart, and brain [27]. In recent years, more and more studies have demonstrated that long-term unhealthy lifestyle habits can lead to intestinal flora imbalance which was considered as a potential inducement for hyperlipidemia [28]. Hence, the stability of the intestinal microbiota and the existence of dominant bacteria play a crucial role in maintaining the normal physiological functions of the intestine. Dietary fiber has the ability to influence the composition of the intestinal microbiota, thus promoting health [29]. Animal and human studies have confirmed that *coix* seed or its extract possesses various pharmacological activities. However, the hypoglycemic and hypolipidemic activity of CSE was still unclear. Therefore, the intervention of CSE on hyperlipidemia mice was conducted to study its effects on glycolipid metabolism.

Our study showed that HFD induced serious inflammatory cell infiltration, hepatocyte balloon-like change as well as lipid droplet accumulation in hepatocytes. CSE intervention significantly relieved these phenomena in a dose-dependent manner. ALT and AST serve as highly sensitive markers for the surveillance of liver function. When the liver cell membrane is damaged, ALT and AST leak into serum resulting in a sharp increase in ALT and AST levels [30]. Our study showed that ALT and AST activities reduced to normal levels after low and high doses of CSE treatment mitigating liver damage induced by HFD. MDA was largely produced in lipid peroxidation which was always accompanied by oxidative stress [31]. Our study indicated that CSE intervention notably alleviated the oxidative stress caused by HFD, especially by decreasing MDA levels. Similarly, *coix* seed treatment significantly improved the levels of GSH and SOD and reduced the content of MDA in brain tissue of stroke model mice [32]. Overall, the findings of this research indicated that CSE was capable of lessening histological damage and averting oxidative damage as well as lipid accumulation in hyperlipidemic mice.

The bodyweight loss after CSE treatment suggested CSE intervention could prevent obesity in hyperlipidemia mice. Lipid biochemical indexes TG, TC, LDL-C, and HDL-C in serum and liver were also notably changed by CSE intervention. TG content in serum significantly increased in HFD mice while notably decreasing to normal level after CSE treatment. However, no significant difference was observed for TG levels in the liver among the four groups. TGs are mainly synthesized and metabolized in the liver for energy storage and supply. In this study, the highest TG level was detected in serum, indicating that blood lipids were the main form of existence in hyperlipidemia mice leading to a high risk of various diseases [33]. TC is an important component in cell membranes and the material for bile acids, vitamin D, and steroid hormone synthesis [34]. In the model and CSE-treated groups, the TC content in the liver decreased significantly, likely because its utilization increased. LD-C and HDL-C are two forms of cholesterol and are directly influenced by TC and TG levels. A marked increase in the LDL-C level in the liver and serum heightens the risks of atherosclerosis, and cardiovascular and cerebrovascular diseases, among others [35]. HDL-C, which is produced in the liver, serves as an anti-atherosclerotic lipoprotein. It functions by transporting cholesterol from extrahepatic tissues to the liver for metabolic processing. Notably, it has an inverse correlation with the risk of multiple diseases [36]. CSE intervention notably decreased the level of LDL-C while increasing HDL-C content in the liver, thereby reducing the risk of the above-mentioned diseases. Meanwhile, FBG and OGTT experiments showed that CSE treatment significantly decreased the glucose level and improved glucose intolerance in hyperlipidemia mice. Our results demonstrated that CSE intervention prevented fat accumulation and thus improved glycolipid metabolism abnormalities. Similar to our study, *coix* seed and probiotic intervention significantly declined the bodyweight, epididymis, and perirenal fat in obese mice [37]. Meanwhile, 18 weeks of zeolite intervention decreased bodyweight and liver coefficients, plasma lipids (TG, TC, LDL-C) levels, and FBG co in high-fat obesity and type 2 diabetes model mice [38].

The metabolic profiles significantly changed in the liver of hyperlipidemia mice after CSE intervention. The top 10 metabolites, including six lipids of PE-NMe (14:0/22:0), LPE (18:1/0:0), LPC (0:0/20:3), FFA (18:4), FFA (18:2), and pentadecanoic acid as well as four amino acids and its metabolites Phe-Gly, tyrosine methylester, alanylphenylalanine, and tyrosylleucine notably decreased after CSE treatment compared to the model group. PE-NMe belongs to the type of glycerophospholipid is a key component of the lipid bilayer in cells involved in metabolism and signal conduction. The decrease in LPE (18:1/0:0) level after CSE treatment reduced lipid formation and altered TG profiles in hepatocytes [39]. This could be one of the factors contributing to the reduction in lipid droplets in the liver. LPC, the hydrolyzed form of PC, is also a key phospholipid component of oxidized low—density lipoprotein (Ox-LDL). Ox-LDL is known to play a significant role in processes such as cell apoptosis, inflammation, and the development of atherosclerosis [40]. The notable decrease in LPC (0:0/20:3) level was consistent with the decline of LDL-C in liver and serum suggesting the improvement of liver condition by CSE treatment. FFA are the products of lipolysis and are mainly used for energy storage. High levels of FFA may lead to oxidative stress and activate signaling pathways that are related to insulin resistance and impaired β cell function. The reduced levels of FFA (18:4) and FFA (18:2) after CSE intervention suggested the alleviation in liver injury and lipid accumulation which was in line with the results of histopathological analysis, red O staining, and oxidative stress. Furthermore, the CSE intervention had an impact on lipid-related pathways, including glycerophospholipid metabolism, α-linolenic acid metabolism, linolenic acid metabolism, and choline metabolisms.

The composition and abundance of gut flora are influenced by various factors including host status, environmental conditions, and dietary [41]. HFD dietary led to the disruption of gut microbiota and increased the number of harmful bacteria leading to chronic, noninfectious, and immune-related diseases, as well as brain pathology, microglial overactivity, and cognitive decline [42,43]. The notably enhanced Shannon index revealed that CSE intervention increased the diversity of gut microbiota. At the phylum level, CSE exposure down-regulated *p_Proteobacteria* level while up-regulated *p_Cyanobacteria* level compared to the model group. So far, a large number of studies have confirmed that intestinal *p_Proteobacteria* could reflect the microecological imbalance or unstable intestinal microbial community structure [44]. Therefore, the decrease in *p_Proteobacteria* was the potential signature for dysbiosis repairment and disease prevention. *p_Cyanobacteria* was reported to negatively correlate with cardiovascular disease [45], cirrhosis [46], and obesity [47]. Therefore, our study suggested that CSE may improve the imbalance of intestinal flora and inhibit the occurrence of diseases like cardiovascular and obesity.

At the genus level, contents of *g_Achromobacter* and *g_Parasutterella* remarkably decreased in CSE-treated groups compared to the model group. *Achromobacter* is mainly isolated from the respiratory tract of cystic fibrosis patients and can trigger a wide variety of infections in hosts [48]. *Parasutterella* is an important gut microbiota with the function of decreasing glucose, producing succinate, and changing the levels of aromatic amino acids, bile acid, and purine [49]. Therefore, in our study, the notable decline of these two microbiota revealed that CSE treatment could increase host immunity as well as inhibit glucose accumulation in hyperlipidemia mice. Moreover, the decrease in *g_Parasutterella* may be the reason for the decrease in aromatic amino acids Phe-Gly, tyrosine methylester, alanylphenylalanine, and tyrosylleucine in this study. Similarly, aloe polysaccharide was also reported to ameliorate obesity-associated cognitive dysfunction in HFD mice by reducing the abundance of *g_Parasutterella* [50]. Parabacteroides, a major component of the human microbiota, has a significant negative correlation with obesity and NFLAD. It plays a role in modulating the inflammatory response and enhancing the integrity of the intestinal barrier [51]. *Anaerotruncus* was reported to be related to cognitive impairment [52]. *Ruminococcaceae* was more prevalent in the gut of lean—phenotype mice compared to obese—phenotype mice. It has been recognized as the most significant biomarker for relieving obesity. This bacterium is positively correlated with the HDL-C content in the liver but negatively correlated with the levels of TC, TGs, and LDL-C [53]. The notable increase in *g_Parabacteroides*, *g_Anaerotruncus*, and *g_unidentified Ruminococcaceae* after CSE intervention suggests its functions of improving intestinal structure, decreasing glucose level, and inhibiting obesity. Water extracts of fermented *Eucommia ulmoides* leaves were also reported to regulate hyperlipidemia-related intestinal flora and metabolites [54]. CSE had a protective effect on liver injury in hyperlipidemia mice through changing metabolic profiles and gut microbiota which were also demonstrated by the results of biochemical indexes and pathological analysis. Consequently, it was hypothesized that CSE might exert an anti-hyperlipidemia effect by regulating the intestinal flora and lipid metabolism.

**Figure 7:**
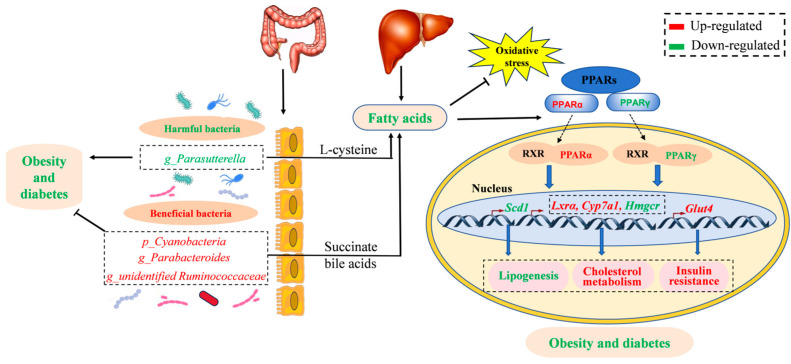
The potential molecular mechanism of CSE anti-hyperlipidemia. CSE ameliorated hyperlipidemia in HFD-induced mice by decreasing the expression levels of fat synthesis genes and increasing the level of lipolysis genes through the PPAR signaling pathway in the liver. In the intestinal tract, SCE decreased the harmful bacteria and increased the beneficial bacteria. Moreover, the metabolites from bacteria of L-cysteine, succinate, and bile acids could cross the intestinal barrier and regulate the PPAR signaling pathway.

According to the outcomes of network pharmacology and molecular docking, the PPAR signaling pathway and its related genes were screened out as the targets for CSE treatment in hyperlipidemia mice. The PPAR family plays an important role in cell signal transduction, metabolic regulation, inflammatory response, and gene expression [55]. The activation of the PPAR pathway is mainly achieved by binding to specific ligands like fatty acids and lipids. *Pparα* regulates the expression of genes involved in lipid metabolism in the liver as well as reduces TG levels in blood [56]. PPARγ is a major regulator for adipocyte differentiation and the abnormality of its content may lead to lipodystrophia [57]. The notable increase in *Pparα* and decrease in *Pparγ* in the liver after CSE intervention suggests that CSE could reduce lipid accumulation in hyperlipidemia mice. *Lxrα* is a key regulatory factor in adipose tissue, which is highly expressed in adipose tissue and participates in lipid metabolism. Studies have shown that *Pparγ* can stimulate the expression of *Lxrα* inhibiting adipose inflammation [58]. The decrease in *Lxrα* in the model group indicates that HFD may lead to inflammation in the liver while CSE treatment improved it to a normal level. As a rate-limiting enzyme in the synthesis of monounsaturated fatty acids, *Scd1* is involved in cellular metabolism and differentiation of precursor fat cells [59]. The notable decrease in *Scd1* level suggested that CSE could reduce fat synthesis. *Glut4* is the direct target regulated by the *Lxr*/*Rxr* heterodimer and the activation of *Lxrα* can up-regulate *Glut4* expression, promote glucose uptake, and improve insulin resistance [60]. The notable raising of *Glut4* indicated that CSE exposure increased the usage of glucose in the liver which was associated with the former results that CSE treatment increased the FBG and OGTT in hyperlipidemia mice. *Hmgcr* is the key gene in the cholesterol synthesis pathway. *Pparα* can decrease the expression of *Hmgcr*, the rate-limiting enzyme in TG synthesis. As a result, it reduces the synthesis of TGs [61]. The remarkable enhancement of *Hmgcr* in a model group led to TG accumulation in the liver and serum while CSE treatment decreased its level to normal which was consistent with the decrease in TGs in serum. *Pparα* regulates its downstream *Cyp7a1* promoting fatty acid β oxidation and bile acid metabolism. In the liver, *Cyp7a1* catalyzes the conversion of cholesterol to 7α-hydroxyl cholesterol, which promotes the breakdown of bile acids [62]. The decrease in *Cyp7a1* in the model group while the increase in the CSE intervention group suggested that CSE reduced lipid accumulation by enhancing the lipolysis process. Therefore, CSE ameliorated hyperlipidemia in HFD-induced mice by decreasing the expression level of fat synthesis genes and increasing the level of lipolysis genes. The potential molecular mechanism of CSE anti-hyperlipidemia is shown in Figure 7.

## 5. Conclusions

Our results suggested that CSE alleviated liver cell injury, lipid droplet accumulation as well as oxidative damage in hyperlipidemia mice. Meanwhile, significantly improved glucose intolerance and reduced bodyweight thereby preventing obesity. Metabolomics analysis revealed that lipid-related metabolites and pathways were highly affected after CSE intervention. 16S RNA sequencing indicated that CSE treatment notably enhanced the diversity of intestinal flora which could inhibit obesity and maintain intestinal balance. Network pharmacology and molecular docking demonstrated that CSE improved hyperlipidemia through the PPAR singling pathway which was then verified by RT-qPCR. The results showed that the expression level of genes from the PPAR pathway notably changed by CSE intervention with lipid synthesis genes significantly declined while lipolysis genes remarkably increased. Our results suggested that CSE could decrease glucose and lipid levels and further alleviate hyperlipidemia through the PPAR signaling pathway.

## Data Availability

The original contributions presented in the study are included in the article/Supplementary Material, further inquiries can be directed to the corresponding author.

## Supplementary Materials

The following supporting information can be downloaded at https://www.mdpi.com/article/10.3390/foods14050770/s1. Table S1 Dietary composition and proportion of HFD group and control group in mice. Table S2. PCR primer sequences. Table S3 Composition analysis of active compounds in CSE (top 10). Table S4. The delta and *p*-values of mice body weight at time 0 and 8 weeks, 8 weeks and 16 weeks. Table S5. The delta and *p*-values of mice food intake at time 0 and 8 weeks, 8 weeks and 16 weeks. Table S6. The delta and *p*-values of mice FBG at time 0 and 4 weeks, 4 weeks and 8 weeks. Table S7. The delta and *p*-values of mice OGTT at time 0 and 60 mins, 60 mins and 120 weeks. Table S8. Information for the active ingredient from *coix* seed. Table S9. Binding energy of active components to target proteins. Table S10. Changes of indexes in mouse model group and CSE treatment group. Figure S1. OPLS-DA score plot. Plots of OPLS-DA score for four different groups (A). OPLS-DA scores and OPLS-DA replacement tests for model vs low dose group (B), and model vs high dose group (C). Figure S2. The heatmaps and linear discriminant analysis (LDA) effect size (LEfSe) analysis. Figure S3. Docking results of Sitosterol α1 in CSE with target proteins.
