# Supplementary material for: Coix Seed Extract Attenuates Glycolipid Metabolism Disorder in Hyperlipidemia Mice Through PPAR Signaling Pathway Based on Metabolomics and Network Pharmacology
Source: Foods. 2025 Feb 24;14(5):770. doi: 10.3390/foods14050770 (PMC11899454; doi:10.3390/foods14050770)
Supplement: Supplementary file 1 [file foods-14-00770-s001.zip › foods-3471903-supplementary.pdf]

Supplemental Data

Coix seed extract attenuates glycolipid metabolism disorder in hyperlipidemia mice through PPAR signaling pathway based on metabolomics and network pharmacology

Min Wang [1, †], Tianming Yang [1, †], Yongjing Xiang [1, †], Junxiao Pang [2], Yao Wang [1], and Dali Sun [1,*]

[1] The Key Laboratory of Environmental Pollution Monitoring and Disease Control, Ministry of Education, School of Public Health, Guizhou Medical University, Guiyang 561113, China
[2] College of Food Science and Engineering, Guiyang University, Guiyang 550005, China
* Correspondence: dalisun11@163.com
† These authors contributed equally to this work.

Supplemental Materials and Methods

S1 Chemical components of the CSE

CSE (20 mg) was weighed added with 500 μL methanol and water mixture with a ratio of 3:1 (*v/v*), vortexed for 30 s, homogenized at 40 Hz for 4 min, sonicated in ice water bath for 5 min, and centrifuged at 12,000 rpm for 15 min. The supernatant was filtered through a 0.22 μm filter membrane. The metabolites were chromatographically detected by an EXION ultra-high performance liquid chromatograph system (UPLC) coupled with a Sciex Qtrap 6500+ (SCIEX Technologies). The mobile was consisted with 0.1% formic acid aqueous solution (A) and acetonitrile (B). Column temperature was set at 40 °C and injection temperature at 4 °C with injection volume of 2 μL. The conditions of mass spectrum were set as follows: ionspray voltage at +5500/－4500 V, curtain gas at 35 psi, temperature at 400 °C, ion source gas at 60 psi, pressure difference at ± 100 V. SCIEX analyst Work Station software (v 1.6.3) was used to collect and process MRM data. Msconventer was applied to convert the MS raw data (wiff) file into TXT format. Internal R programs and databases were used for peak detection and annotation.

Supplemental Tables

Table S1 Dietary composition and proportion of HFD group and control group in mice.

group	Diet composition and proportion
Control	60% corn<br>20% soybean meal<br>5% wheat sub-meal<br>5% fish meal<br>5% soybean oil<br>2% limestone powder<br>2% calcium carbonate<br>0.5% vitamins<br>0.5% mineral elements
HFD	49% basal diet<br>20% fructose<br>12% casein<br>10% lard<br>3% sesame oil<br>2% calcium bicar-bonate<br>2% experimental animal premix<br>1.5% cholesterol<br>0.5% sodium cholate

Table S2. PCR primer sequences.

Genes	Forward primer（5′− 3′）	Reverse primer（5′− 3′）
β-actin	GGCTGTATTCCCCTCCATCG	CCAGTTGGTAACAATGCCATGT
PPARα	AACATCGAGTGTCGAATATGTGG	CCGAATAGTTCGCCGAAAGAA
PPARγ	GGAAGACCACTCGCATTCCTT	GTAATCAGCAACCATTGGGTCA
LXRα	CTGATTCTGCAACGGAGTTGT	GACGAAGCTCTGTCGGCTC
SCD1	TTCTTGCGATACACTCTGGTGC	CGGGATTGAATGTTCTTGTCGT
GLUT4	ACACTGGTCCTAGCTGTATTCT	CCAGCCACGTTGCATTGTA
HMGCR	AGAGCGAGTGCATTAGCAAAG	GATTGCCATTCCACGAGCTAT
CYP7A1	GCTGTGGTAGTGAGCTGTTG	GTTGTCCAAAGGAGGTTCACC

Table S3 Composition analysis of active compounds in CSE (top 10).

Number	Retention time (min)	Exact mass	Formula	Identification	Category
1	13.03	282.25588	$C_{18}H_{34}O_2$	oleic acid	fatty acyls
2	2.82	165.0790	$C_9H_{11}NO_2$	L-phenylalanine	amino acid and derivatives
3	0.69	129.078979	$C_6H_{11}NO_2$	L-pipecolic acid	amino acid and derivatives
4	0.55	160.07356	$C_7H_{12}O_4$	pimelic acid	fatty acyls
5	0.56	129.042594	$C_5H_7NO_3$	5-oxoproline	amino acid and derivatives
6	12.92	256.24023	$C_{16}H_{32}O_2$	palmitic acid	lipids
7	1.82	131.094629	$C_6H_{13}NO_2$	6-aminocaproic acid	fatty acyls
8	0.93	117.078979	$C_5H_{11}NO_2$	L-valine	amino acid and derivatives
9	12.93	414.386165	C29H50O	beta-sitosterol	steroids and steroid derivatives
10	13.12	412.370515	C29H48O	stigmasterol	steroids and steroid derivatives

Table S4 The delta and P-values of mice body weight at time 0 and 8 weeks, 8 weeks and 16 weeks.

group	body weight /(g)			
	$\Delta_{\text{week 8-0}}$	$p_{\text{week 8-0}}$	$\Delta_{\text{week 16-8}}$	$p_{\text{week 16-8}}$
Control	8.95±0.42	-	-1.54±0.78	-
Model	7.83±0.88	0.116	-3.77±1.08	0.044^{*}
Low	7.02±1.33	0.075	-8.53±0.70	0.0001^{****}
High	7.36±2.08	0.264	-6.02±3.05	0.07

Note: * indicate the significance between CSE treated group with control group. $^{*}P<0.05$, $^{****}P<0.0001$.

Table S5 The delta and P-values of mice food intake at time 0 and 8 weeks, 8 weeks and 16 weeks.

group	Food intake /(g/d/mice)			
	Δ week 8-0	P week 8-0	Δ week 16-8	p week 16-8
Control	0.54±0.69	-	0.78±1.35	-
Model	-0.06±0.59	0.317	-0.10±0.53	0.352
Low	0.09±0.64	0.454	-0.05±0.08	0.349
High	-0.88±0.93	0.100	0.47±0.58	0.736

Table S6 The delta and P-values of mice FBG at time 0 and 4 weeks, 4 weeks and 8 weeks.

group	FBG /(mmol/L)			
	$\Delta_{\text{week 4-0}}$	$p_{\text{week 4-0}}$	$\Delta_{\text{week 8-4}}$	$p_{\text{week 8-4}}$
Control	-0.23±4.05	-	0.07±3.44	-
Model	-3.43±0.21	0.244	2.90±0.79	0.237
Low	-4.53±2.06	0.177	3.70±1.21	0.160
High	-7.83±3.43	0.068	5.63±2.4	0.083

Table S7 The delta and P-values of mice OGTT at time 0 and 60 mins, 60 mins and 120 weeks.

group	OGTT /(mmol/L)			
	$\Delta_{min\ 60\text{-}0}$	$p_{min\ 60\text{-}0}$	$\Delta_{min\ 120\text{-}60}$	$p_{min\ 120\text{-}60}$
Control	5.80±3.46	-	-5.87±6.27	-
Model	1.83±2.29	0.173	1.07±3.44	0.168
Low	4.60±7.43	0.812	-1.90±10.97	0.615
High	6.07±3.83	0.933	-4.80±3.99	0.816

Table S8 Information for the active ingredient from *coix* seed.

MOL ID	Molecule Name	MW	OB (%)	DL
MOL001323	Sitosterol α1	426.80	43.28	0.78
MOL001494	Mandenol	308.56	42.00	0.19
MOL002372	(6Z,10E,14E,18E)-2,6,10,15,19,23-Hexamethyltetracosa-2,6,10,14,18,22-hexaene	410.80	33.55	0.42
MOL002882	[(2R)-2,3-dihydroxypropyl] (Z)-octadec-9-enoate	356.61	34.13	0.30
MOL000359	sitosterol	414.79	36.91	0.75
MOL000449	Stigmasterol	412.77	43.83	0.76
MOL008118	Coixenolide	591.08	32.40	0.43
MOL008121	2-Monoolein	356.61	34.23	0.29
MOL000953	CLR	386.73	37.87	0.68

Table S9 Binding energy of active components to target proteins.

Active ingredient	Binding energy/(Kcal·mol^{-1})							
	RXRα	AR	NR1H3	CYP19A1	PPARδ	PPARα	SCD	RXRγ
Sitosterol α1	-5.21	-5.0	-5.12	-3.78	-6.78	-5.6	-5.64	-2.94
CLR	-4.45	-4.69	-4.29	-4.53	-5.16	-5.54	-5.2	-4.14
Sitosterol	-4.30	-5.24	-4.44	-4.12	-5.50	-4.29	-5.39	-3.05
Stigmasterol	-4.71	-4.77	-4.43	-3.96	-5.15	-5.01	-4.18	-3.53

Table S10 Changes of indexes in mouse model group and CSE treatment group.

Index name	Model VS CSE	
	Up-regulated	Down-regulated
Liver weight/body weight	↑	-
Serum TG	-	↓
Serum LDL-C	-	↓
Liver TC	↑	-
AUC	-	↓
AST	-	↓
ALT	-	↓
MDA	-	↓
Pparα	↑	-
Pparγ	-	↓
Scd1	↑	-
Hmgcr	-	↓
Lxrα	↑	-
Cyp7α1	↑	-
Glut4	↑	-
g_Parasutterella	-	↓
p_Cyanobacteria	↑	-
g_Parabacteroides	↑	-
g_unidentified Ruminococcaceae	↑	-

Supplemental Figures

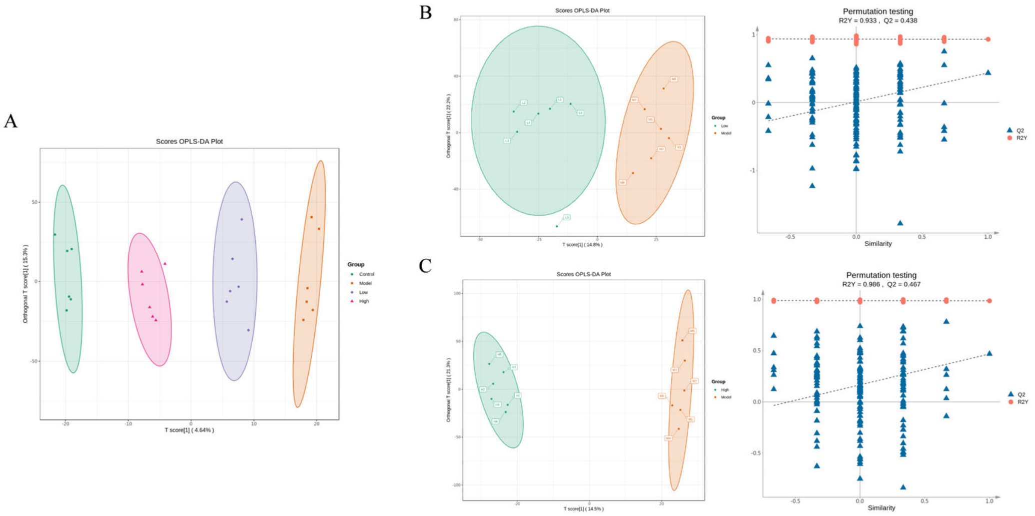

Figure S1 OPLS-DA score plot. Plots of OPLS-DA score for four different groups (A). OPLS-DA scores and OPLS-DA replacement tests for model *vs* low dose group (B), and model *vs* high dose group (C).

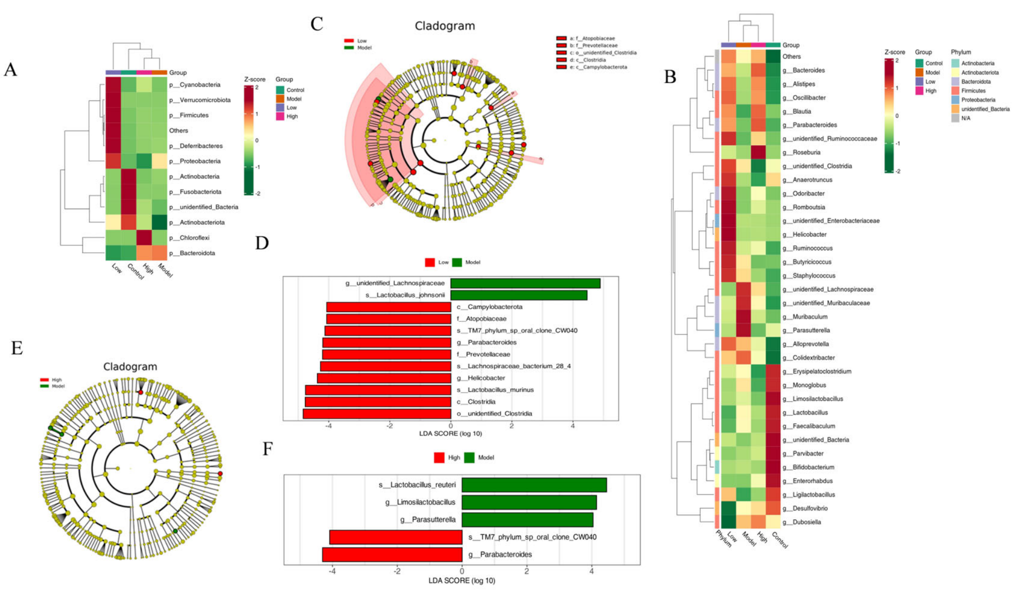

Figure S2 The heatmaps and linear discriminant analysis (LDA) effect size (LEfSe) analysis. The heatmaps of gut microbiota at phylum (A) and genus levels (B), Cladogram and LEfSe for model *vs* low dose group (C, D) and model *vs* high dose group (E, F).

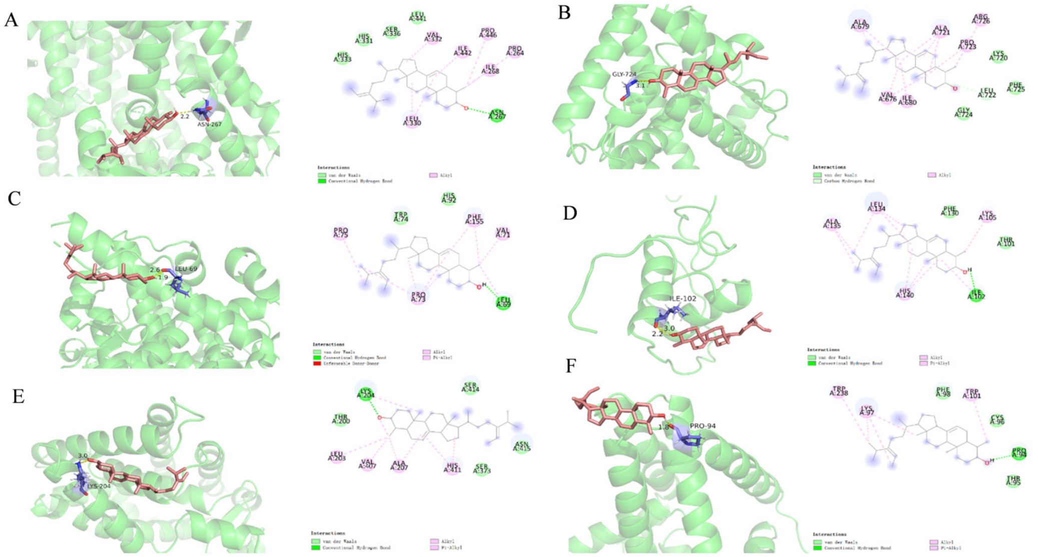

Figure S3 Docking results of Sitosterol α1 in CSE with target proteins. Sitosterol α1-RXRα (A), Sitosterol α1-AR (B), Sitosterol α1-NR1H3 (C), Sitosterol α1-PPARδ (D), Sitosterol α1-PPARα (E), Sitosterol α1-SCD (F).
